# Clinical application of the Hybrid Assistive Limb (HAL) for gait training—a systematic review

**DOI:** 10.3389/fnsys.2015.00048

**Published:** 2015-03-25

**Authors:** Anneli Wall, Jörgen Borg, Susanne Palmcrantz

**Affiliations:** ^1^Department of Rehabilitation Medicine, Danderyd University HospitalStockholm, Sweden; ^2^Department of Clinical Sciences, Karolinska InstituteStockholm, Sweden

**Keywords:** rehabilitation, robotics, gait, walking, locomotion, paresis, review, gait machine

## Abstract

**Objective:** The aim of this study was to review the literature on clinical applications of the Hybrid Assistive Limb system for gait training.

**Methods:** A systematic literature search was conducted using Web of Science, PubMed, CINAHL and clinicaltrials.gov and additional search was made using reference lists in identified reports. Abstracts were screened, relevant articles were reviewed and subject to quality assessment.

**Results:** Out of 37 studies, 7 studies fulfilled inclusion criteria. Six studies were single group studies and 1 was an explorative randomized controlled trial. In total, these studies involved 140 participants of whom 118 completed the interventions and 107 used HAL for gait training. Five studies concerned gait training after stroke, 1 after spinal cord injury (SCI) and 1 study after stroke, SCI or other diseases affecting walking ability. Minor and transient side effects occurred but no serious adverse events were reported in the studies. Beneficial effects on gait function variables and independence in walking were observed.

**Conclusions:** The accumulated findings demonstrate that the HAL system is feasible when used for gait training of patients with lower extremity paresis in a professional setting. Beneficial effects on gait function and independence in walking were observed but data do not allow conclusions. Further controlled studies are recommended.

## Background

Normal gait depends on the functional integrity and interactions in sensory-motor neural networks at spinal and supraspinal levels (Bowden et al., 2013). This complex system may be disturbed in many neurological conditions such as stroke or spinal cord injury (SCI) resulting in limited mobility and impaired gait function, which are major challenges in neuro rehabilitation. Intensive, repetitive task specific training may drive beneficial neuroplasticity, enhance functional restitution and improve final outcome (Kwakkel et al., 2004; Langhorne et al., 2009, 2011; Peurala et al., 2014). However, there is a need for further development of training methods in response to an increasing understanding of the individual capacity for regaining functioning (Krakauer et al., 2012; Bowden et al., 2013).

Approaches to improve gait function after stroke and SCI include treadmill training with or without use of partial body weight support (BWS), yet the evidence to support this is inconclusive (Schwartz and Meiner, 2013; Dobkin et al., 2014). Gait machines (GM) may allow more reproducible gait movements compared to conventional training and reduce the burden on the therapist. GM work according to the end-effector principle (foot plates move the feet in a controlled gait pattern) or as exoskeletons, which have joints matching the limb joints and motors that drive movements over these joints to assist, e.g., leg movements (Hesse et al., 2010). A recent Cochrane review concluded that electromechanically assisted gait training in combination with physiotherapy after stroke increases the odds of achieving independent walking and most so when applied for severely impaired patients in the first 3 months after stroke (Mehrholz et al., 2013) but less clear after SCI (Mehrholz et al., 2012).

The importance of incorporating more active participation than allowed by gait machines to enhance training effects and the need for new concepts and devices are recognized (Dobkin, 2009; Pennycott et al., 2012). One new approach is represented by the Hybrid Assistive Limb system (HAL). HAL is an exoskeleton with a hybrid system allowing both a voluntary and an autonomous mode of action to support training of gait. HAL comprises a control algorithm and supporting devices, where each knee and hip joint can be controlled separately. Key features of the HAL system have been reported in detail (Kawamoto, 2002; Suzuki et al., 2007; Kawamoto et al., 2010). Movements are triggered by use of either the “Cybernic Voluntary Control” (CVC), which is based on the users voluntary activation of gait muscles as recorded by surface electromyography (EMG), or by the “Cybernic Autonomous Control” (CAC), which is based on the users weight shifting and input from force pressure sensors in the shoes. The CVC mode allows the operator to adjust the degree of support for each joint and reduce the support as training progress and to adjust settings to achieve a gait pattern that is as close as possible to normal gait. In case of complete loss of voluntary activation of gait muscles the CAC mode may be used. Gait is then initiated and sustained by input from force-pressure sensors in the shoes. HAL is manufactured in single-leg and double-leg versions and training with HAL may be performed with or without BWS.

A number of clinical studies with HAL have been conducted and there is a need for an evaluation of available data to guide further trials. The aim of this report was to provide a systematic review in order to evaluate current evidence with regard to feasibility (i.e., usability and safety) and effects and to make recommendations for further studies.

## Methods

**Figure 1:**
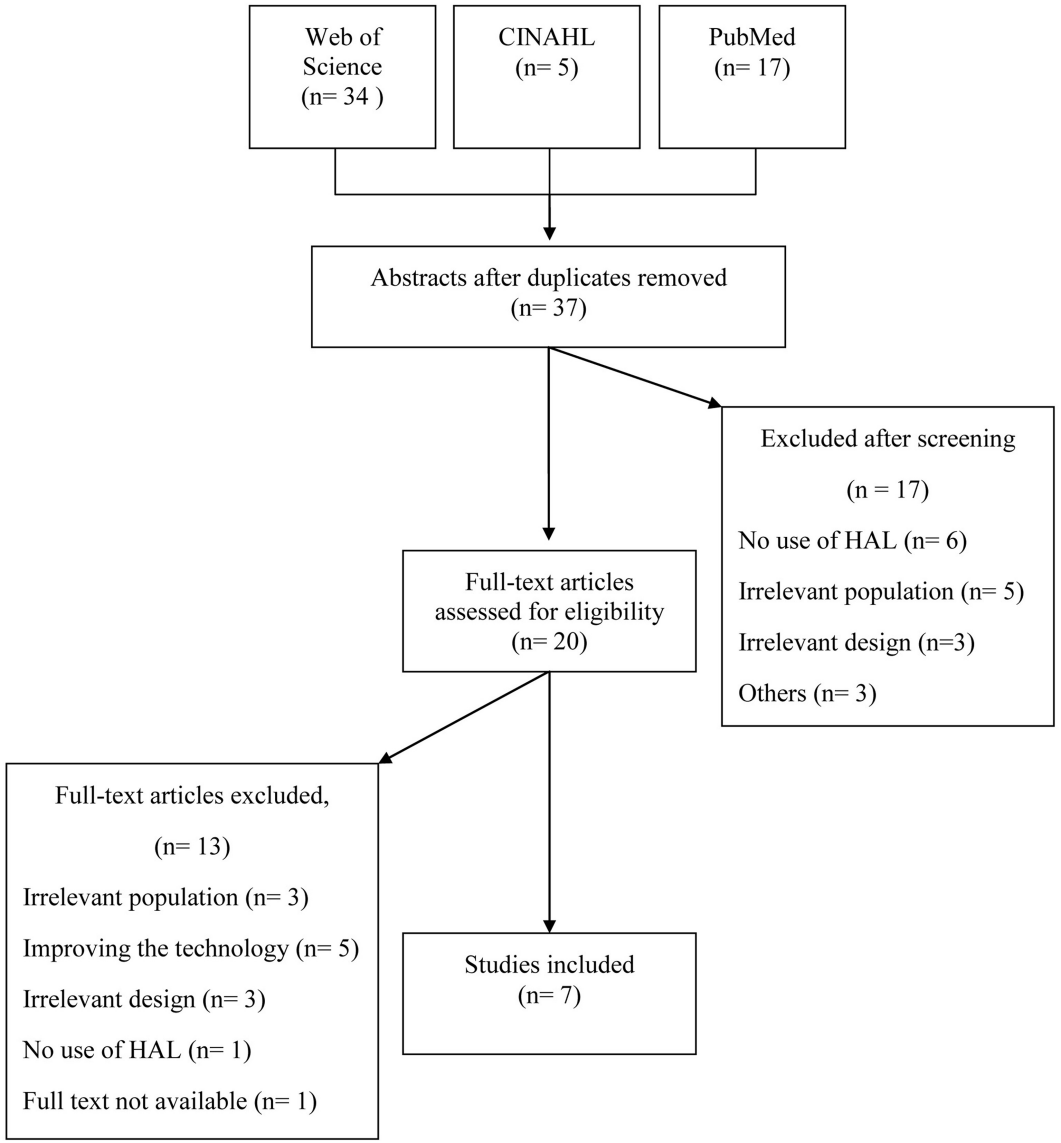
**Presentation of the results of the systematic search of the literature**.

A systematic search of the literature was conducted using the databases Web of Science, PubMed, and CINAHL. Both MeSH (Medical Subject Headings for Medline) terms and free text relevant for the subject were used and detected synonyms were added to the search. Search terms were (MeSH terms in bold): ((((robot OR robots OR robotic OR robotics OR robot-assisted OR exoskeleton OR machine-assisted OR electro-mechanic OR DGO OR “driven gait orthosis”))) AND (gait OR gaits OR walking OR walk OR walks OR locomotion OR “motor activity”)) AND (HAL OR “hybrid assistive limb” OR “wearable robot”). Search limitations were “Humans” and “English,” while publication date was unlimited. Using the same search terms, a search was also performed at clinicaltrials.gov, in order to identify ongoing studies and/or unpublished papers (Clinicaltrials, online). Abstracts identified were screened and studies were considered relevant if they addressed any clinical application of the HAL system regardless of study design. If needed the full text article was retrieved and assessed. Relevant studies were exported to EndNote where duplicates were identified and removed. Reference lists of these studies were manually searched for further articles. Studies were included if they were primary research articles, concerned gait training with the Hybrid Assistive Limb. Studies only reporting technology data, including only healthy subjects, single subjects or reviews were excluded. Thirty-seven articles were identified, 20 were retrieved in full text for assessment of eligibility and 13 of these did not fulfill inclusion criteria (see Figure 1). Overall 7 studies met the inclusion criteria and were subject to data extraction and analyses (Maeshima et al., 2011; Kawamoto et al., 2013; Kubota et al., 2013; Ueba et al., 2013; Aach et al., 2014; Nilsson et al., 2014; Watanabe et al., 2014). Included studies were subject to critical review by two independent reviewers.

The quality of the included studies, regarding risk of confounding and bias, was evaluated in accordance with the Scottish Intercollegiate Guidelines Network (SIGN) criteria (SIGN, online^1^). According to SIGN the methodological quality can be coded (++) meaning *all or most of criteria fulfilled*, (+) *some of the criteria fulfilled* or (−) *few or no criteria fulfilled*. The evaluation was performed independently by two investigators and in case of disagreement, a third reviewer was consulted. Since two investigators are authors of one of the included studies (Nilsson et al., 2014) this study was assessed by a fourth investigator.

Data extraction was performed by one investigator and checked by the two additional investigators. The extracted data comprised; characteristics of participants, intervention protocols and settings, outcome measures and effects and feasibility.

## Results

The 7 studies included 8–38 participants each, 6 were single group studies and 1 was a randomized controlled trial (RCT). In total, the studies involved 140 participants of whom 118 completed the intervention protocols and 107 used HAL for gait training. Extracted study data and results of the quality evaluation are presented in the Table 1. All included studies were found to have a high risk of bias and confounding according to the SIGN criteria and do not provide data for a meta-analysis.

**Table 1:** **Extracted study data**.

**First author**	**Kawamoto et al., 2013**	**Maeshima et al., 2011**	**Nilsson et al., 2014**	**Ueba et al., 2013**	**Watanabe et al., 2014**	**Aach et al., 2014**	**Kubota et al., 2013**
Aim	To investigate the feasibility of locomotor training with the HAL in chronic stroke patients and to examine differences between two functional ambulation subgroups	To evaluate the effects of the HAL suit on the gait of stroke patients undergoing rehabilitation	To explore the safety and feasibility of the HAL system when used for early onset, intensive gait training as part of an inpatient rehabilitation program for patients with hemiparetic stroke	To investigate the feasibility and safety of the HAL suit in the rehabilitation of patients in the acute phase after stroke	To examine the effects of gait rehabilitation using the HAL for patients in the recovery phase of stroke to test the feasibility of intervention and outcome assessment protocols for a future randomized controlled trial on a larger scale	To evaluate the possibilities of exoskeletal locomotor training with HAL under voluntary control and identify beneficial effects on functional mobility of the patients Hypothesis: that exoskeleton treadmill training is feasible and safe in application and capable of improving ambulatory mobility in chronic SCI patients	To investigate the feasibility of 16-session (8 week) HAL rehabilitation training for patients with limited mobility
Study design and setting	Single group study Out-patient setting	Single group study Inpatient rehabilitation setting	Single group study Inpatient rehabilitation setting	Single group study Inpatient rehabilitation setting	Randomized controlled study (outcome assessment not blinded) Inpatient rehabilitation setting	Single group study Out-patient setting	Single group study Out-patient setting
Inclusion criteria	1. Requirement of physical assistance or assistive devices for standing up sitting down, and/or walking; 2. Understanding study protocol and expressing voluntary consent 3. A body shape that could fit in the robotic suit HAL 4. Concurrent use of physical and occupational therapies	Not reported	1. <7 weeks since stroke 2. Able to sit 5 min 3. Unable to walk due to paresis 4. Postural control to allow standing with assistance 5. Understand instructions 6. Express informed consent 7. Body size that fits HAL	1. Hemiplegi or ataxia after stroke 2. Height >120 cm weight <100 kg 3. Glascow coma scale >9 4. Systolic BP 100–160 mmHg 5. Saturatio*n* > 90% 6. Heart rate between 40 and 120 beats/min 7. Body temperature <37.5°C	1. Hemiparesis from unilateral ischemic/hemorrhagic stroke 2. Time since onset <6 months	1. Traumatic SCI with cronic incomplete or complete paraplegia 2. Present motor functions of hip and knee extensor and flexor muscle groups	1. Musculoskeletal disability affecting ambulation 2. Physical assistance or assistive devices in at least one of following daily activities: standing up, sitting down, and walking 3. Ability to understand study and to express consent 4. Body size that fit HAL 5. Ability to undergo usual physical and occupational therapies
Diagnosis and number of patients	Stroke Divided into two subgroups based on FAC-score FAC 2–3 (dependent ambulatory) FAC 4–5 (independent ambulatory) Included *n* = 16 Completed *n* = 16	Stroke Patients were divided in 3 groups: 1. Assisted group 2. Handrail group 3. Quad cane group included *n* = 16 completed *n* = 16	Stroke Included *n* = 8 Completed *n* = 8	Stroke Included *n* = 22 Completed *n* = 16	Stroke Included *n* = 32 Completed *n* = 22 (11 in each group)	SCI Included *n* = 8 Completed *n* = 8	Stroke *n* = 12 SCI *n* = 8 Musculoskeletal diseases *n* = 4 Other diseases *n* = 14 Included *n* = 38 Completed *n* = 32
Time since event/disease onset	Months, mean: 47.1 ± 37.6 Months, range: 13–132	Days, mean: 52 Days, range: 29–116	Days, range: 6–46	Days, mean: Group 1: 12.7 ± 7.6 Group 2: 9.5 ± 2.4 (divided into two groups based on occurrence of orthostatic hypotension)	Days, mean: HAL group 58.9 ± 46.5 Control group 50.6 ± 33.8	Months, mean: 97.2 ± 88.4 Years, range: 1–19	Years, range: 1–54
Age	Years, mean: 61 Years, range: 18–84	Years, mean: 63 Years, range: 53–78	Years, mean: 53 Years, range: 39–64	Years, mean: 66.6 Years, range: ?-90 (min not reported)	Years, mean: HAL group 67 Control group 75.6 Years, range: not reported	Years, mean: 48 Years, range: 36–63	Years, mean: 53.2 Years, range: 18–81
Gender	Men *n* = 12 Women *n* = 4	Men *n* = 9 Women *n* = 7	Men *n* = 8 Women *n* = 0	Men *n* = 7 Women *n* = 15 (included, completed not reported)	Men *n* = 11 Women *n* = 11 HAL group, men *n* = 7, women *n* = 4 Control group, men *n* = 4, women *n* = 7	Men *n* = 6 Women *n* = 2	Men *n* = 21 Women *n* = 11
Intervention	HAL-training: with use of mobile suspension system and harness. Sit-to-stand and walking No of sessions: *n* = 16 Times/week: approximately *n* = 2 (individualized) Duration, weeks, mean: 10.8 ± 3.5 HAL version: double leg Training lasted 90 min/session HAL training was 20–30 min/session	HAL-training: walking (overground), stair climbing No of sessions: *n* = 1 Times/week: NA Duration: NA HAL version: double leg	HAL-training: with use of treadmill and body weight support No of sessions: median 16, range 6–31 Times/week: *n* = 5 Duration, weeks: approximately *n* = 3 (individualized) HAL version: double leg Training lasted 90 min/session, max 60 min (effective time)	HAL-training: not reported No of sessions, mean: 3.8 ± 3.1 Times/week: not reported Duration, days, mean: 12.1 ± 7 HAL version: double leg	HAL training: with use of mobile suspension system and harness if necessary HAL group: no of sessions: *n* = 12 Times/week: *n* = 3 Duration, weeks: *n* = 4 HAL version: single leg Control group receiving conventional training: no of sessions: *n* = 12 Times/week: *n* = 3 Duration, weeks: *n* = 4 Training lasted 20 min per session	HAL-training: with use of treadmill and body weight support. Included some regular physiotherapy No of sessions, mean: 51.75 ± 5.6 Times/week: *n* = 5 Duration, days: *n* = 90 HAL version: double leg Training lasted 90 min/session	HAL-training: with use of mobile suspension system and harness, or treadmill and body weight support No of sessions: *n* = 16 Times/week: *n* = 2 Duration, weeks: *n* = 8 HAL version: double leg Training lasted 90-min/session (including all). Net walking time was approximately 20 min
Assessments	10m walk test^*^ (walk speed, number of steps, cadence) Timed up and go Berg balance scale	10m walk test (stride length, speed) Physiological cost index Assessed before, during, after HAL training and the next day	10m walk test Timed up and go Berg balance scale Functional ambulation categories NIH stroke scale Fugl-Meyer-LE Clinical outcome variable scale Falls-efficacy scale Barthel index Functional independence measure EQ-5D	Orthostatic hypotension and several medical and functional variables were assessed	10m walk test (Max walking speed) Timed up and go Functional ambulation categories^*^ 6 min walk test Fugl-Meyer-LE Short physical performance battery Isometric muscle strength	10m walk test (time, number of steps, assistance) Timed up and go 6 min walk test Walking index for SCI II (WISCI II) Lower extremity motor score Muscle volume ASIA impairment scale	10m walk test^*^ Timed up and go^*^ Berg balance scale^*^
Results	Dependent ambulators: improvements in 10m walk test (speed, cadence and number of steps) and berg balance scale. statistically significant differences (*p* < 0.05) Independent ambulators: improvements in Berg Balance Scale. Statistically significant differences (*p* < 0.05) Whole group: improvements in 10m walk test and berg balance scale. Statistically significant differences (*p* < 0.05) Adverse events reported as: no training-related serious adverse events were observed	During HAL training physiological cost index (PCI) increased in *n* = 11 participants. PCI was associated with ability to ambulate (*p* < 0.05) Walking speed decreased during training in *n* = 12 participants. Speed was associated with ability to ambulate (*p* < 0.05) Stride length increased during training in *n* = 4 participants. Stride length was not associated with ability to ambulate (*p*-value not reported) Adverse events: not reported	Improvements in FAC, Fugl-Meyer-LE, 10m walk test, and berg balance scale Statistically significant differences: not reported Adverse events reported as: no serious adverse events occurred	Improved walking and torso posture *n* = 2 Standing with HAL *n* = 12 No change *n* = 2 Withdrew *n* = 6 Statistically significant differences (*p* < 0.05): not reported Adverse events reported as: orthostatic hypotension (*n* = 4)	HAL group: improvements in independent walking (FAC), walking speed, timed up and go, 6 min walk test and Fugl-Meyer LE. Statistically significant differences (*p* < 0.05) Control group: improvements in independent walking (FAC), short physical performance and timed Up and go. Statistically significant differences (*p* < 0.05) Between groups: improvements in independent walking (FAC) greater in HAL group. Statistically significant differences (*p* < 0.04) Adverse events reported as: no participants withdrew because of adverse effects	All patients improved in treadmill training with HAL. Mean walking speed and average walking time increased Improvements in 10m walk test (gait speed, number of steps), lower extremity motor score, timed up and go, 6 min walk test statistically significant differences (*p* < 0.05) Improvements in walking index for SCI II. No statistically significant differences (*p* < 0.05) Adverse events reported as: neither adverse nor severe adverse events occurred during the intervention	Improvements in gait speed, steps and cadence based on 10m walk test, (27 participants) Statistically significant differences (*p* < 0.05) Improvements according to timed up and go and berg balance scale. No statistically significant differences (*p* < 0.05) Adverse events reported as: no serious training-related adverse events
Quality according to SIGN criteria	(−)	(−)	(−)	(−)	(−)	(−)	(−)

* *Indicated as the primary outcome in the study*.

### Characteristics of participants included in the studies

The most frequent diagnosis reported was stroke (*n* = 106) followed by complete or incomplete SCI (*n* = 16) and other disorders (*n* = 18). Five of the studies included solely persons with stroke, 1 study included solely persons with SCI (Aach et al., 2014) and 1 study included persons with stroke (*n* = 12) and SCI (*n* = 8) as well as other disorders (*n* = 18) (Kubota et al., 2013). Mean age of the participants ranged from 48 to 67 years with a total range of 18–90 years.

In 6 of the included studies a total of 65.7% (*n* = 67) of the participants who completed the study intervention were men and 34.3% (*n* = 35) were women. Ueba et al. (2013) did not report gender for participants completing the study but among the included 32% (*n* = 7) were men and 68% (*n* = 15) were women.

The total time from disease onset to inclusion ranged from 6 days to 54 years. For persons with SCI the time from injury ranged from 1 to 19 years and for persons with stroke this time ranged from approximately 6 days to 16 years. Three studies (Maeshima et al., 2011; Nilsson et al., 2014; Watanabe et al., 2014) included persons early after stroke, with a mean range of 31–59 days since stroke onset. Two other studies included persons later than 1 year and up to 16 years after stroke onset (Kawamoto et al., 2013; Kubota et al., 2013).

The number of reported dropouts during the study interventions ranged from 6 to 10 and was 22, in total (18 with stroke, 1 with SCI and 3 participants with other diagnosis,). Reported reasons for dropouts were medical (*n* = 5), technical (*n* = 1), discharge (*n* = 2), personal (*n* = 4) and withdrawal of consent (*n* = 4). Another 6 participants dropped out due to inappropriate size of shoes, lumbar spondylosis which prevented correct fitting and/or depressive status.

### Intervention protocols and settings

All except 1 (Ueba et al., 2013) of the 7 studies specifically addressed gait training. The HAL training protocols showed a great variation with regard to frequency, intensity and number of sessions performed. Most studies applied HAL training ≥2 times per week during ≥4 weeks with durations of ≥20 min per session. In the studies involving persons with stroke, the total number of sessions per participant training with HAL ranged from 1 (Maeshima et al., 2011) to 31 (Nilsson et al., 2014). In studies involving persons with SCI, Aach et al. (2014) used a mean of 51.75 sessions while the number of sessions for persons with SCI in the study by Kubota et al. (2013) was 16. Data on the use of the active CVC mode and the autonomous CAC mode was not consistently reported. Based on the information provided, we anticipate that 6 of the studies used the CVC mode during training but the extent is not clear. One study (Ueba et al., 2013) did not report on modes used. Four studies reported the total length of each training sessions to be approximately 90 min including assessments, donning, doffing and effective walking time. The effective training time in these studies was approximately 20–30 min per session. Training with HAL was performed by use of BWS and/or a mobile suspension system in 5 studies, by over ground training in 1 study and was not defined in 1 study. Four studies were conducted in inpatient rehabilitation settings and 3 in out-patient care (see Table 1).

### Outcome measures and effects

All assessments were performed without wearing HAL except for one study (Maeshima et al., 2011) where measurements were performed both with and without HAL. Outcome measures mainly related to walking ability. Most frequently used were the 10 m walking test (*n* = 6) (Wade et al., 1987), Timed up and Go (*n* = 5) (Podsiadlo and Richardson, 1991), and Berg Balance Scale (*n* = 3) (Berg et al., 1992). Assessments performed at baseline and after the training period were reported in all studies except 1 (Ueba et al., 2013). No study reported on a long-term follow up. The explorative RCT (Watanabe et al., 2014) compared the effect of HAL-training to the effect of conventional training in the subacute phase after stroke and included 11 participants in each group. The study shows a significant differences (*p* = 0.04) according to the Functional Ambulation Categories (FAC) (Holden et al., 1984) between groups, in favor for the HAL training group. This study has several limitations with regard to study sample size, varying time after stroke and lack of blinding of outcome assessments, as recognized by the authors. One other study (Nilsson et al., 2014) also used FAC and observed improvements suggesting a beneficial effect in a single group. Other studies also report on varying effects such as improvements in walking- and torso posture, gait speed, number of steps and cadence, functional ambulation/independent walking, motor function in lower extremity, activity performance and/or balance (see Table 1).

### Adverse events

All studies except 1 (Maeshima et al., 2011) explicitly reported on adverse events. Except for transient complaints related to pressure of the suit, irritated skin, training related pain etc., no adverse events during training with HAL were reported.

## Discussion

The aim of this review was to explore existing evidence regarding gait training with the exoskeleton HAL (Hybrid Assistive Limb). We included 7 studies, each with small study samples but comprising a total of 140 patients. Of these, 118 completed the intervention and 107 used HAL. Studies differed in terms of aim, design, duration of intervention, patients/diagnosis, setting and participant characteristics as well as allocation, randomization, blinding and outcome measures. Only 1 study compared training with HAL with other training (Watanabe et al., 2014) but outcome assessment in that study was not blinded. Although no study provides conclusive data on the effects of gait training with HAL as compared to other training and the risk of confounding and bias was considered high, the experiences of training with HAL and the responses that were observed will be useful in the design of further studies.

### Feasibility

In total, adult subjects within a broad age range (18–90 years) participated. Both genders were represented, however two thirds were men. Since the majority of subjects had a stroke diagnosis and gender proportions are fairly similar in this diagnostic group, the uneven distribution is surprising but only scarcely commented on in the studies.

Study participants in both post-acute and long-term after stroke onset were represented and 88 out of 106 included subjects completed the study interventions. One small experimental study included subjects with paraplegia 1–19 years after spinal cord injury where all completed the intervention (Aach et al., 2014) while no study addressed effects of training with HAL in the post-acute phase after SCI. The severity of paresis and gait problems varied both within and between the included studies, from severe (only able to maintain sitting balance) to moderate (independent walkers) and a corresponding use of HAL mode and BWS. Reasons for training of independent walkers were not stated or discussed in the included studies although plausible beneficial effects may be, e.g., an increase in walking speed and/or distance or level of independence. The reported numbers of dropouts were low and a total of 107 participants, representing a broad spectrum of motor impairments completed >1500 training sessions with the HAL system without any reported serious adverse events.

Thus, the accumulated results of the included studies demonstrate that training with the HAL system is feasible when applied in professional settings, irrespective of the patients age and sex and the severity of the lower extremity paresis. The feasibility of training with HAL in the post-acute phase after SCI will need to be explored further.

### Intervention protocols and settings

Even though not stated, the variability in applied frequency, intensity and duration of the reported training sessions and evaluations of outcome, probably reflect both theoretical and practical considerations of, e.g., training needed to achieve significant effects, participants' functional level and study resources. Reasonably, the optimal design would allow training programs to be on the edge for each participant's capacity with regard to the intensity and length of each training session. The intensity and length of the training periods must also consider the patient's functional level as well as the capacity and aims with regard to, e.g., on neuroplasticity, musculoskeletal function, cardiovascular function, gait pattern or independence in walking. Three or more sessions weekly during the training period would probably be justified from a neuroplasticity and relearning perspective (Bowden et al., 2013).

Both single- and double-leg versions of HAL were used even though not specified in all studies. Reasonably the double-leg version is most relevant for subjects with paraparesis and the single-leg version most often appropriate for subjects with one sided paresis.

Only 2 of the studies included patients early after stroke when the potential to utilize beneficial neuroplasticity processes is higher (Bowden et al., 2013) and there is a need for controlled HAL studies in this area. Post-acute studies are more challenging as they have to consider both the impact of spontaneous recovery early after the event as well as other post-acute health problems.

### Outcome measures and effects

Outcome measures in the included studies primarily relate to aspects of gait function and walking. Most frequently used was the 10 m walking test, which is a measure of over ground walking speed. Six studies report a positive impact on gait function after HAL training, 5 of these specifically on walking speed and 2 studies report increased level of independence in walking according to the FAC (Nilsson et al., 2014; Watanabe et al., 2014). FAC is the most commonly used outcome measure in studies of walking after robotic training for patients with severe to moderate walking limitations in both the acute and chronic phase after stroke (Geroin et al., 2013). FAC takes the persons level of independence and amount of personal assistance required into account, which from the individuals' perspective is more important than walking speed. However, gait speed may be associated with functional ambulation ability (Perry et al., 1995; Dobkin et al., 2014) and a gait speed improvement may generate improved function and quality of life (Schmid et al., 2007). Therefore, we suggest both the FAC and the 10 m walking test to be used in further studies. In studies including participants with severely impaired gait function at baseline and who are unable to walk 10 m, the 2 min walk test (Kosak and Smith, 2005) is an option to be considered in order to achieve baseline data also when participants cannot walk (i.e., 0 m in 2 min). The potential effect of HAL training on movement related function such as gait pattern is poorly addressed in the included studies. Future studies should consider using assessments that cover also these aspects, for example by use of 3-dimensional motion analysis.

Data on self-perceived aspects of the training were scarce and we found no data on perceived activity performance, participation in everyday life, health or cost-effectiveness that need to be approached in future studies. Further, no study reported on potential effects on cardiovascular, metabolic, emotional or cognitive functions of training with the HAL system. The possible additional value of training with HAL in these areas should be of interest in future studies.

Further, controlled studies should compare training with the HAL system with the most relevant alternative training method. As pointed out, gait machines such as the Lokomat differs from the HAL system in terms of the degree of active patient participation. Comparison studies of these gait machines would be of interest. However, until now, studies using Lokomat have not consistently demonstrated effects, regarding sensory-motor function, gait speed, balance and/or mobility, that are superior to those achieved with conventional training (Swinnen et al., 2010; Ucar et al., 2014; van Nunen et al., 2014) although there might be other advantages such as less therapist burden. Thus, further studies that compare the effects of training with the HAL system to the effects of well designed “conventional training” are justified. Moreover, studies combining robotics with other therapeutic interventions with increasing evidence support, such as Fluoxetine (Chollet et al., 2011), BMI (Brain-Machine Interface) (Shindo et al., 2011; Noda et al., 2012) or brain stimulation (Liew et al., 2014) are also of great interest.

Currently, there are a number of exoskeletons at various stages of development or clinical applications. In addition to differences in mechanical design and control strategies existing exoskeletons uses different activation systems to produce movement of the limb. The most common are hydraulic, pneumatic, and electric motor actuator. In a recent review by Chen et al. (2013) focused on lower extremity robots, the authors divides exoskeletons in different subgroups depending on their functioning and design and conclude that real-time control strategies with timely assistance are a new promising area in rehabilitation therapy. The importance of incorporating more active participation in electromechanical gait training (Dobkin, 2009; Pennycott et al., 2012) as well as of allowing variation of the task during training to promote adequate motor learning (Hidler and Sainburg, 2011) has also been addressed previously.

Some recently developed designs of exoskeletons have taken this into account by establishing intention-based control strategies. In the Ekso (Ekso Bionics, online^2^), ReWalk (Rewalk, online^3^), and in Indego (Indego, online^4^) stepping is initiated by shifting of bodyweight. In Indego, shifting of body weight is used in combination with functional electric stimulation (FES). Another exoskeleton the MINDWALKER (Mindwalker, online^5^) uses EEG and EMG based control.

Recent studies using Ekso for patients in different stages after SCI conclude that the system is safe and show improvements in walking while wearing Ekso (Ekso Clinical Research, online^6^). For inpatient rehabilitation after stroke the authors find Ekso safe to use and indicate that the training may have an effect on cadence as a result of training with Ekso (Ekso Clinical Research, online). However, the number of participant in these studies are limited and do not allow any further conclusions. Ongoing studies after both SCI and severe stroke (Clinicaltrials, online) will evaluate potential effects on ambulation and mobility.

Indego has been introduces in single-subject clinical trials in SCI patients (Quintero et al., 2011; Farris et al., 2014). An ongoing study will evaluate the safety and effectiveness of using Indego for non-ambulatory or poorly ambulatory SCI patients during standing and walking (Clinicaltrials, online^7^).

In a study by Zeilig et al. (2012) ReWalk was found to be well tolerated and did not cause any adverse events among persons with SCI. This was repeated in a study by Spungen et al. (ReWalk Peer Reviewed Publications, online^8^) where persons with motor-complete paraplegia performed different community-based activities while wearing ReWalk. Several ongoing studies where ReWalk is used after SCI are registered at Clinicaltrials.gov.

Like the HAL, exoskeletons such as the powered knee-ankle-foot-orthosis (KAFO) (Sawicki and Ferris, 2009) and NEUROExos (Cain et al., 2007) use EMG activity to detect a person's intended movement. In both KAFO and NEUROExos EMG activity is used to control the activation of a pneumatic power system to provide torque over the ankle and/or knee joint. In HAL the wearer's joint torque is estimated from the EMG signals on both hip and knee muscles and an electrical motor actuator is used to generate power over these joints (Suzuki et al., 2007).

This brief survey of other exoskeletons points to a general need for randomized controlled studies where exoskeletons that allow active participation are compared to other types of interventions as well as for studies with larger study populations.

### Study limitations

Since the Hybrid Assistive Limb was developed and is most frequently used in Japan there might be studies published in Japanese journals that were not included in this review. Of note, in 6 of the included studies, the inventor and CEO of the company, Professor Y. Sankai, behind the Hybrid Assistive Limb system is one co-author. No studies using qualitative approach were discovered, which could have been due to the search strategy used. However, even when a broader search was performed, no qualitative studies appeared.

## Conclusions and recommendations

This review identified consistent evidence that the use of the HAL system is feasible when used for gait training in hospital and rehabilitation settings. Data suggest that such training may have beneficial effects on gait function and independence in walking after stroke and after spinal cord injury, but do not allow any conclusions in this respect. Further, well designed controlled studies in these areas are recommended to explore effect sizes and to be followed by larger, confirmatory studies.

### Conflict of interest statement

The authors declare that the research was conducted in the absence of any commercial or financial relationships that could be construed as a potential conflict of interest.

## Footnotes

^1^SIGN. Scottish Intercollegiate Guidelines Network. Available online at: http://www.sign.ac.uk/methodology/checklists.html [Accessed Dec 2014].

^2^Ekso Bionics. Available online at: http://intl.eksobionics.com/ekso. [Accessed March 2015].

^3^ReWalk. Available online at: http://www.rewalk.com/products/rewalk-rehabilitation/. [Accessed March 2015].

^4^Indego. Available online at: http://www.indego.com/indego/en/home. [Accessed March 2015].

^5^Mindwalker. Available online at: https://mindwalker-project.eu/. [Accessed March 2015].

^6^Ekso Clinical Research, Summary of Findings. Available online at: http://intl.eksobionics.com/clinical-research-summary-download. [Accessed March 2015].

^7^Clinicaltrials.gov. Available online at: https://www.clinicaltrials.gov/ [Accessed Dec 2014].

^8^ReWalk, Peer Reviewed Publications. Available online at: http://www.rewalk.com/technology/peer-reviewed-publications-copy/. [Accessed March 2015].
